# Collaborative Robotic Wire + Arc Additive Manufacture and Sensor-Enabled In-Process Ultrasonic Non-Destructive Evaluation

**DOI:** 10.3390/s22114203

**Published:** 2022-05-31

**Authors:** Rastislav Zimermann, Ehsan Mohseni, Momchil Vasilev, Charalampos Loukas, Randika K. W. Vithanage, Charles N. Macleod, David Lines, Yashar Javadi, Misael Pimentel Espirindio E Silva, Stephen Fitzpatrick, Steven Halavage, Scott Mckegney, Stephen Gareth Pierce, Stewart Williams, Jialuo Ding

**Affiliations:** 1 Centre for Ultrasonic Engineering, University of Strathclyde, Glasgow G1 1XW, UK; ehsan.mohseni@strath.ac.uk (E.M.); momchil.vasilev@strath.ac.uk (M.V.); charalampos.loukas@strath.ac.uk (C.L.); randika.vithanage@strath.ac.uk (R.K.W.V.); charles.macleod@strath.ac.uk (C.N.M.); david.lines@strath.ac.uk (D.L.); yashar.javadi@strath.ac.uk (Y.J.); 2 Advanced Forming Research Centre, University of Strathclyde, Renfrew PA4 9LJ, UK; misael.pimentel@strath.ac.uk (M.P.E.E.S.); s.fitzpatrick@strath.ac.uk (S.F.); steven.halavage@strath.ac.uk (S.H.); scott.mckegney@strath.ac.uk (S.M.); 3 Welding Engineering and Laser Processing Centre, University of Cranfield, Cranfield MK43 0AL, UK; s.g.pierce@strath.ac.uk (S.G.P.); s.williams@cranfield.ac.uk (S.W.); jialuo.ding@cranfield.ac.uk (J.D.)

**Keywords:** non-destructive evaluation, in-process robotic NDE, Wire + Arc Additive Manufacture (WAAM), ultrasound testing, total focusing method

## Abstract

The demand for cost-efficient manufacturing of complex metal components has driven research for metal Additive Manufacturing (AM) such as Wire + Arc Additive Manufacturing (WAAM). WAAM enables automated, time- and material-efficient manufacturing of metal parts. To strengthen these benefits, the demand for robotically deployed in-process Non-Destructive Evaluation (NDE) has risen, aiming to replace current manually deployed inspection techniques after completion of the part. This work presents a synchronized multi-robot WAAM and NDE cell aiming to achieve (1) defect detection in-process, (2) enable possible in-process repair and (3) prevent costly scrappage or rework of completed defective builds. The deployment of the NDE during a deposition process is achieved through real-time position control of robots based on sensor input. A novel high-temperature capable, dry-coupled phased array ultrasound transducer (PAUT) roller-probe device is used for the NDE inspection. The dry-coupled sensor is tailored for coupling with an as-built high-temperature WAAM surface at an applied force and speed. The demonstration of the novel ultrasound in-process defect detection approach, presented in this paper, was performed on a titanium WAAM straight sample containing an intentionally embedded tungsten tube reflectors with an internal diameter of 1.0 mm. The ultrasound data were acquired after a pre-specified layer, in-process, employing the Full Matrix Capture (FMC) technique for subsequent post-processing using the adaptive Total Focusing Method (TFM) imaging algorithm assisted by a surface reconstruction algorithm based on the Synthetic Aperture Focusing Technique (SAFT). The presented results show a sufficient signal-to-noise ratio. Therefore, a potential for early defect detection is achieved, directly strengthening the benefits of the AM process by enabling a possible in-process repair.

## 1. Introduction

In 2019, the global metal Additive Manufacturing (AM) market size was valued at 2.02 billion € and was predicted to grow by up to 27.9% annually until 2024 [1]. AM technology plays a critical role in the latest industrial revolution, Industry 4.0, where there is a demand for smart factories capable of fabricating high-quality customized products [2]. One such AM technology, called Wire + Arc Additive Manufacturing (WAAM), is a rapidly developing metal AM technology, based on a directed energy deposition process [3], which promises an automated fabrication of structurally complicated three-dimensional (3D) near-net shaped components [4]. The process is aiming to achieve superior cost-efficiency by reducing energy usage, material waste and time as compared to traditional subtractive manufacturing methods [5]. The technology has attracted the attention of sectors such as aerospace and naval engineering, due to their interest in weight reduction and increased geometrical complexity of solid metal components [6]. Moreover, WAAM offers the potential to build products using otherwise expensive materials such as titanium alloys, steel or nickel-based super-alloys [4].

Conventionally, the quality assurance of WAAM is performed by Non-Destructive Evaluation (NDE) after the full built completion via manually deployed methods such as ultrasound testing [7], eddy-current [8] or X-ray based imaging [9]. These techniques, however, require complex and time taking manipulation between workstations and often pre-processing or machining of the components [9], affecting the production throughput and therefore overall cost if a defect is discovered. Hence, in order to maintain the benefits of the already highly automated WAAM process, the demand for automatically deployed flexible NDE integrated in-process is high [10]. 

The detection of defects, in-process, facilitates the potential for real-time repair or early scrapping of parts, preventing the manufacturer from time-taking deposition of costly material over defective layers. Moreover, the deployment of automated NDE offers greater benefits such as positional accuracy, repeatability and high rates of inspection as compared to human NDE operators [11]. 

Recently published research has presented an important advancement in the field of automated in-process NDE of arc-based welding processes [12,13,14], which are manufacturing methods with similar applicable attributes and challenges to WAAM. The development of a multi-robot welding cell demonstrated the possibility of robotic welding and automated ultrasound NDE [15]. Full automation was achieved by a novel sensor-enabled robotic system based around a real-time embedded controller which enabled: (a) real-time communication, (b) data acquisition and (c) control of the process. Moreover, a UDP (User Datagram Protocol) communication protocol established through the Robot System Interface (RSI) [16], developed by industrial robot manufacturer KUKA, was used for the sensor-based robotic motion correction that could influence the pre-programmed robot’s path through the sensor’s feedback on the fly. The motion corrections were executed based on a novel developed motion software operating in real-time intervals (4 milliseconds intervals for KUKA Robot Controller (KRC) 4). Therefore, it was reported possible to utilize the cell for automated ultrasound inspection in three modes: (1) post-process continuous, (2) inter-pass in-process or (3) live-arc in-process. Further, the use of Force-Torque (FT) sensor-driven robotic motion for automated NDE of complex geometries was explored in [10]. The FT sensor facilitated path correction required for contact-based scanning of the aircraft wing cover through an as-built surface geometry that was inconsistent with geometry in the original CAD model. Therefore, the research faced similar automation challenges, associated with transducer deployment on the estimated pre-programmed path, applicable to possible automated NDE deployment on near net-shaped WAAM.

The ultrasound-based in-process NDE of welds at elevated temperatures was made possible by the development of novel, phased array ultrasound transducer (PAUT)-based, high-temperature and dry-coupled roller-probes [15,17]. Thanks to its design, based on a PAUT coupled through a water delay line and a flexible silicone rubber, the roller-probe was reported capable of withstanding temperatures of up to 350 °C, which made this device well suited for in-process NDE of arc-based manufacturing processes, where the resistance to elevated temperatures is highly desired [17]. This was a significant advancement in ultrasound NDE transducer development, given a typical commercial PAUT can only operate up to around 60 °C, while commercial delay lines can offset this limit to the temperatures only up to around 150 °C for a short period of time [18]. 

Further, the roller-probe technology has also been developed to couple with an as-built surface of WAAM components without the use of liquid couplants [19]. However, when considering the roller-probe inspection approach, new challenges emerged, as the as-built WAAM component features a non-flat and varying surface geometry (in both the scanning and the traverse direction) resulting in high contact forces being required to assure full compliance of the roller-probe tyre to the surface. Hence, the design must facilitate the transmission of the maximum possible ultrasonic energy without suffering signal losses. This resulted in the alteration of the internal liquid delay line with a heat-resistant solid core (delay line) made of Polyetherimide Polymer. 

The key advantage of the novel WAAM roller-probe NDE approach is the removal of the post-deposition processing stages, which often included waiting for the sample to cool down, machining operations and manipulation between workstations. The inspection would then be performed through a flat surface either using direct gel-coupled contact with the sample or in water immersion tanks using gantry systems [7,20,21].

Owing to the novel WAAM dry-coupled roller-probe ultrasound NDE concept, the research has presented a possibility to detect Lack of Fusion (LoF) defects as small as 5 × 0.5 × 0.5 mm (width, length and height), through an as-built surface of the WAAM wall [22]. The ultrasound data acquisition called Full Matrix Capture (FMC) enabled the collection of raw time-domain data without consideration of any refractive boundaries or couplant conditions as the imaging was executed at the post-processing stage [23]. The developed ultrasound post-processing algorithms, based on the Synthetic Aperture Focusing Technique (SAFT) and Total Focusing Method (TFM) made it possible to overcome complications associated with multiple refractions present at a non-flat surface of WAAM and internal components of the roller-probe [24]. These algorithms, also called the SAFT-TFM package, were based on Delay and Sum (DaS) computational logic, where at first the Time of Flight (ToF) elapsed, between a PAUT element and a targeted image pixel, was calculated. Subsequently, the signal response from the corresponding time sample of an elementary A-scan was summed to the pixel. ToF calculation was repeated for every transmit–pixel–receive combination, thus, a fully focused image of the WAAM interior was formed. This novel ultrasound NDE approach was, however, presented on static inspection of WAAM components and was not yet deployed in-process on high-temperature builds. 

Therefore, in this paper, the authors present an experimental multi-robot cell designed for WAAM deposition and automated in-process dry-coupled ultrasound NDE using a custom WAAM roller-probe. Within the cell, the plasma-arc WAAM process was controlled by the deposition software while a full external control of the NDE process was achieved by the sensor-enabled adaptive motion control package adapted to in-process WAAM NDE. The automated high-temperature WAAM roller-probe was deployed within a dwell time, set for inter-layer cooling, while sufficient coupling with the as-built surface of WAAM during the inspection was assured by the FT sensor. In this work, a titanium WAAM straight component (wall) with embedded tungsten reflectors was deposited to evaluate the performance of the in-process NDE approach. The use of tungsten tubes as cylindrical artificial reflectors, with known diameter, for ultrasound inspection technique calibration and evaluation has found its application in the fields of in-process welding inspection [12,13] as well as ultrasound inspection of WAAM [25]. An advantage of the tungsten can be realized by the possibility to manufacture inclusions, closely simulating defects such as Lack of Fusion (LoF) or keyholes, at the desired location [26]. During the in-process NDE, the position encoded FMC data were acquired using a high-speed ultrasound phased array controller. The SAFT-TFM package, then, enabled the highly accurate detection of artificial reflectors presented on an amplitude C-scan image. C-scan imaging provided a top-view over an interior of the WAAM component and was found effective for data review from a large inspection volume [27]. Thus, for the first time, a volumetric in-process ultrasound NDE of as-built WAAM was achieved, directly supporting research on producing right-first-time WAAM parts.

## 2. The Architecture of the WAAM + NDE Cell 

### 2.1. Hardware 

**Figure 1:**
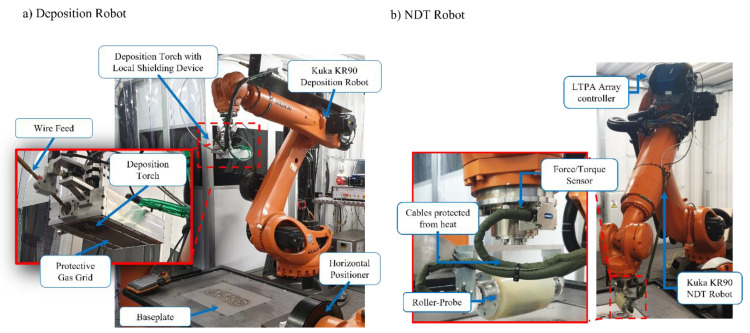
Implemented (**a**) WAAM deposition cell with plasma arc process, and (**b**) Roller-Probe based NDE.

The automated robotic WAAM and NDE system depicted in Figure 1 was designed based on 2 × 6 Degrees of Freedom (DoF) industrial robotic manipulators (KUKA KR90 R3100) employed as a WAAM deposition robot and as an inspection robot. Additionally, as a part of the deposition robot, a horizontal rotary positioner (KUKA DKP-400V3) was also located within this cell and utilized as a rotational tooling mainframe and substrate clamping device. The deposition hardware, physically mounted on a deposition robot’s end-effector, featured a water-cooled plasma-arc welding torch (controlled by: EWM-TETRIX 552 AC/DC SYNERGIC PLASMA AW welder) integrated into a deposition device with a local shielding [28], as seen in Figure 1a. The local shielding device was an aluminum enclosure with multiple gas outlet channels fitted, that provided an additional supply of the argon shielding gas on a high-temperature WAAM to prevent atmospheric contamination that could result in oxidation of the fresh deposit. Further, a wire-feed outlet with adjustable height was fitted on the deposition device, positioned to supply feedstock into the melt pool. The wire supply was controlled by an automatic wire feeder (EWM T drive 4 Rob 3 Li, EWM) that was attached directly to the deposition robot’s arm as well. Lastly, the deposition head was equipped with a high dynamic range welding camera (Xiris XVC-1000) used to remotely assess the deposition quality.

**Figure 2:**
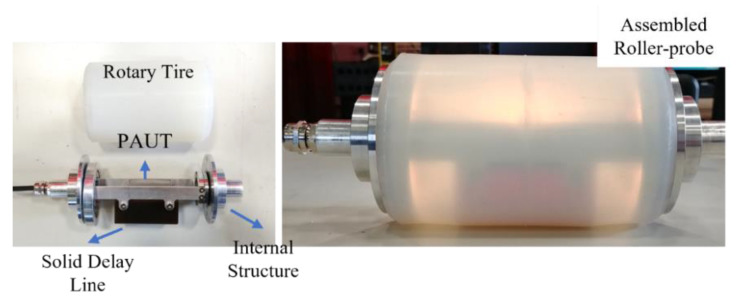
The internal structure of the roller-probe (**left**) and assembled device (**right**).

An inspection robot, seen in Figure 1b was equipped with an FT sensor (FTN-GAMMA-IP65 SI-130-10, Schunk, Germany) mounted on the end effector. A WAAM roller-probe was, then, attached to an FT sensor serving as an end effector to the robot flange. The roller-probe, depicted in detail on Figure 2, was driven by a high-speed phased array ultrasound controller LTPA (PEAK NDT, United Kingdom) mounted directly on the robot arm. Further, the communication between all hardware was achieved by a network switch (Zyxel Gigabit ethernet switch) enabling control of the WAAM process and NDE via a single ethernet connection plugged into the PC. 

### 2.2. Software Setup 

#### 2.2.1. Deposition

In this work, the deposition robot was controlled by a pre-installed PC with a WAAMCtrl (WAAM3D, Milton Keynes, UK) [29] application, streaming the deposition commands (robot paths, deposition parameters) directly to the deposition robot via RSI over an ethernet connection. The tool-path plan was generated using WAAMPlanner Software (WAAM3D, UK) [30], where the desired component was imported as a Computer-Aided Design (CAD), sliced into layers according to the pre-defined layer height, segmented into a set of individual building blocks from which the series of tool-paths was generated. Depending on the variables, such as material, geometry or deposition process, the deposition parameters were given to a WAAMPlanner and the post-processed file was generated, translating the information to a ready-to-stream xml file. 

#### 2.2.2. NDE Software

**Figure 3:**
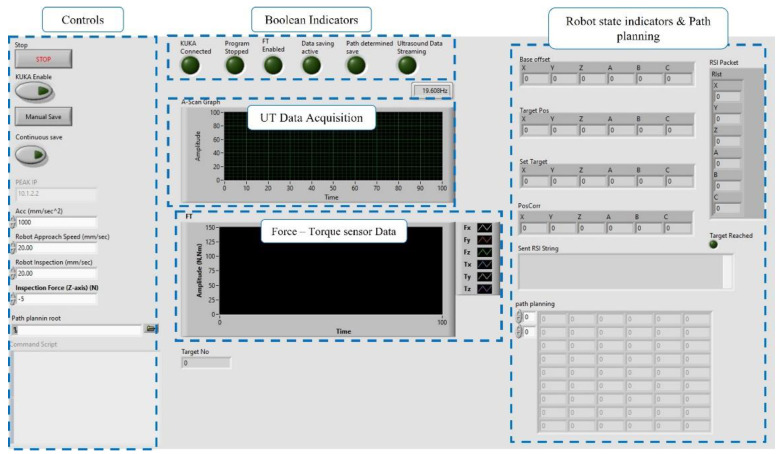
LabVIEW GUI for NDE process control and monitoring.

The NDE inspection was guided by a software platform developed in the LabVIEW programming environment [31], which offers reliable communication between instruments and fast prototyping, through several available toolboxes and libraries. The Graphic User Interface (GUI) is presented in Figure 3, where the platform consisted of parallel state machines responsible for executing the program in sequence, controlling the inspection robot kinematics through the FT sensor feedback and ultrasound data acquisition in real-time. 

The real-time corrections (every 4 milliseconds) of the robot’s motion, based on linear interpolation, and control used for the in-process NDE work were based on a flexible robotic motion framework presented in [15] and developed for in-process inspection and automated NDE purposes. During the inspection, real-time adjustments of the inspection robot velocity, acceleration and contact force were available. Position-determined triggers were implemented to automatically switch between inspection and travel speed of the inspection robot, enabling/disabling the FT sensor-driven motion and data acquisition when needed. The *Z*-axis force control through the FT sensor was used to maintain sufficient contact with the WAAM component while the operator maintained the ability of real-time adjustments of the kinematics. In this work, the *Z*-axis motion corrections, associated with maintaining a steady force at the inspection speed, were calculated by the KRC based on the RSI configuration diagram. The X and Y translation, and A, B and C rotation-axes motion correction always remained in control of the initial inspection path-planning, while the appropriate motion corrections were calculated within the developed motion framework in 4 ms intervals and streamed through the RSI. 

Further, taking the advantage of real-time communication with the inspection robot, the timestamped position of the inspection robot during an inspection was encoded to each FMC frame acquired. The FMC data were then processed within a MATLAB environment using a SAFT-TFM algorithm package, enabling positionally accurate analysis. 

## 3. Experimental WAAM Manufacturing

### 3.1. WAAM Wall Path Planning and Deposition Parameters

To demonstrate the WAAM and NDE cell concept, and evaluate its performance, a titanium (Ti-6Al-4V) WAAM wall was chosen and designed for fabrication. The experimental wall was set to be 300.0 mm in length, 25.0 mm wide and a height given as 25.0 mm. However, knowing the nature of the WAAM process delivering near net-shaped components [4], extra material volume post-deposition was expected. Moreover, the height of the wall was not considered important since the goal was to evaluate the inspection of WAAM’s interior with a specific volume. Therefore, the built process was stopped when the wall was found sufficiently high for in-process NDE demonstration to be performed.

**Figure 4:**
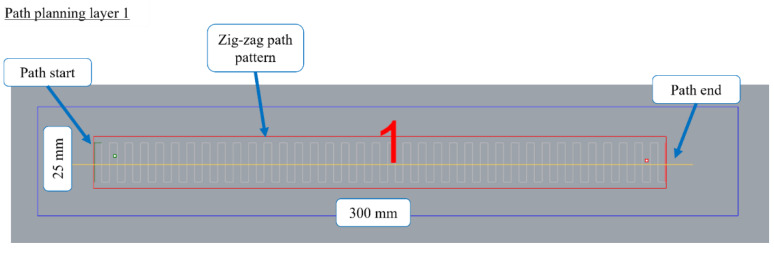
Deposition Path Planning for Layer 1 of an experimental WAAM wall.

The path planning designed in WAAMPlanner, seen in Figure 4, consisted of an oscillating deposition strategy [32], where a single bead, with a square zig-zag pattern, was deposited per layer. Relevant deposition parameters can be seen in Table 1 below. 

### 3.2. WAAM Wall Deposition

**Figure 5:**
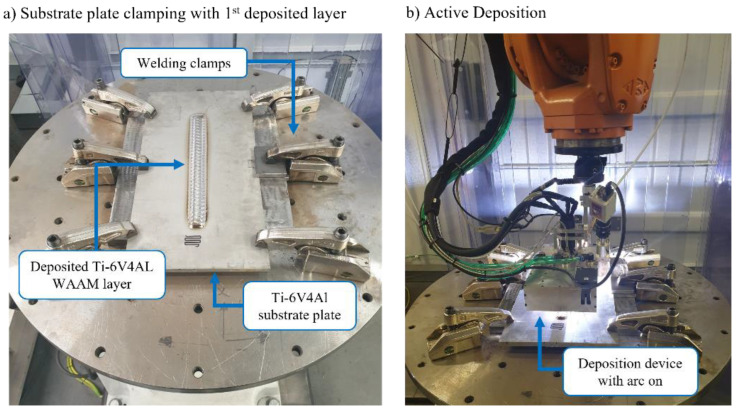
Deposition clamping setup and a substrate plate with a deposited 1st layer (**a**) and deposition process with an active torch (**b**).

Figure 5a displays a deposition setup where an experimental wall was built on a Ti-6V-4AL substrate plate, 12.0 mm thick, clamped to the tooling which was mounted on a rotary table of the horizontal positioner. The plate was clamped using welding clamps to prevent bending caused by heat-induced residual stress [33], typical for arc-based manufacturing processes such as welding [34]. 

This clamping set-up has created a challenging and restricting working envelope; hence, the first stage of manufacturing was calibration and verification of the path motion by a dry run. At this step of WAAM part fabrication, the robot traveled through the produced deposition paths without an active torch or wire feed. Therefore, the correct positioning of the robot could be assured, knowing that the deposition head would not collide with the clamping. This was extremely important, especially during the deposition of the first few layers, after which the deposition head was high enough not to collide with welding clamps. 

Following, Figure 5b shows an active deposition of the 1st layer, while the completed pass on the substrate plate is visible in Figure 5a image. It is worth mentioning, that the height of the first layer was measured to be 3.5 mm. 

### 3.3. Ultrasound Reflector Planting

**Figure 6:**
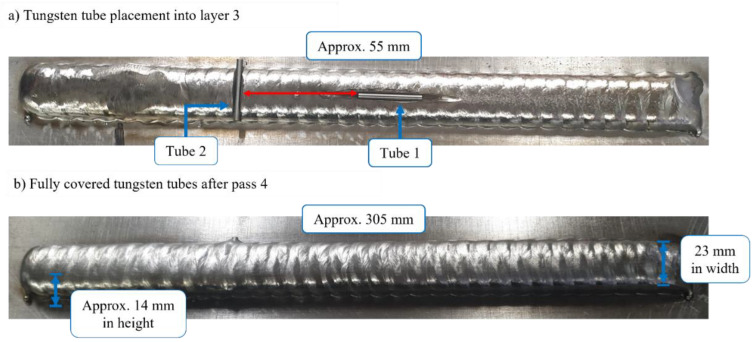
Tungsten tube embedding into layer 3 (**a**) and a subsequently deposited layer 4 covering tubes (**b**).

To evaluate the NDE defect detection capability, artificial reflectors were embedded into the experimental wall. In this work, tungsten tubes with parameters specified in Table 2 were utilized for this purpose. Two tubes were embedded into layer 3 by producing slots using a portable grinding machine. The tubes were located approximately 55 mm from each other. Tube 1 was placed parallel to the wall, in the approximate centre of the bead. Tube 2, on the other hand, was embedded in the transverse direction to the wall as seen in Figure 6a. 

Further, Figure 6b depicts the wall after layer 4 where the tungsten rods were fully covered by the freshly deposited titanium. No significant inconsistencies (defects) in the surface quality that could cause a potential failure of the building process were observed once layer 4 was completed. However, a minor material built up was observed which was corrected after the subsequent layer deposition.

## 4. In-Process NDE of the Experimental WAAM Wall

### 4.1. Ultrasound Inspection Parameters

The ultrasound data were acquired using a roller-probe featuring a solid delay line housed in a silicone rubber tyre. The PAUT, with specifications found in Table 3, was positioned to sit on the top of the delay line. 

The FMC data were collected using an LTPA phased array controller with 200 V excitation voltage and a fixed hardware gain of 60 dB. The time-domain matrix of the signals was formed by 3000 data samples for each transmits–receive pair at a sampling frequency of 50 MHz. During the data post-processing stage, the following acoustic velocities for longitudinal ultrasound waves were used for refraction and time-of-flight computations: (1) Delay line = 2480 m/s, (2) Rubber = 1006 m/s and (3) Titanium = 6100 m/s. These values were obtained by ultrasound pulse-echo measurements of the individual roller–probe’s components and titanium coupons cut from a previous trial and heated to 150 °C. 

### 4.2. In-Process NDE

To demonstrate the ultrasound in-process NDE capability on the titanium wall with embedded tungsten tubes, producing an air gap inside the WAAM, two subsequent layers were deposited to build a six-layer-high component. The deposition of two additional layers enabled a natural surface profile common for plasma WAAM deposition [4], without any significant negative influence from previous grinding and tungsten tube embedding. 

**Figure 7:**
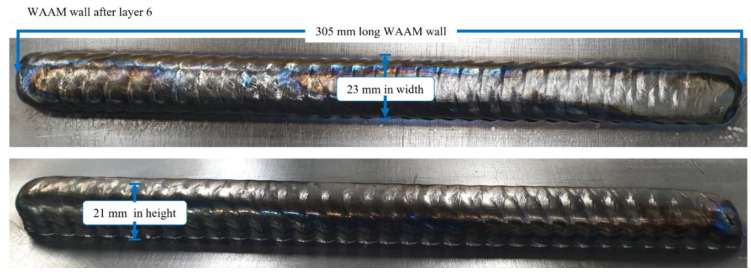
Completed experimental wall and its dimensions.

Figure 7 shows a completed deposition of the experimental wall after layer six with a measured height of approximately 21 mm with an average layer height of approximately 3.5 mm. A width of 28 mm and a length of 305 mm were also measured.

As suggested by the literature [32], there is an optimal inter-pass dwelling time to allow for cooling of Ti-6Al-4V WAAM built using the oscillation deposition strategy. Therefore, the in-process NDE can be integrated into the build process to leverage this inter-pass dwelling time to complete the inspection of the last pass without delaying the built process. Accordingly, a dwell time of 9 min was set to allow inter-pass cooling during the deposition of the experimental wall as suggested by [35]. This time was set to avoid the formation of coarse $\alpha_{GB}$ phase grain microstructure, and thus, achieve optimal mechanical properties of this hypothetical component. Moreover, the time was found sufficiently long for in-process NDE to be performed without causing costly delays in the production process. 

Before starting the NDE, the surface temperature was taken using a handheld thermometer. The surface temperature of the WAAM was measured and ranged to be between 180–230 °C along the wall, which was much lower than the operational limit of the roller-probe (resistant up to 350 °C).

**Figure 8:**
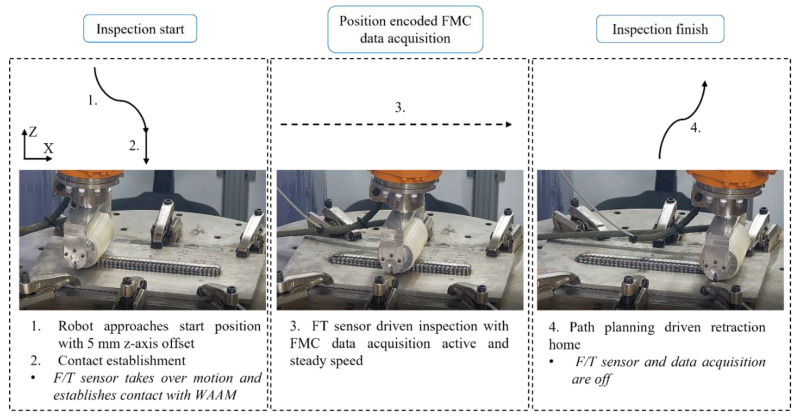
Inspection diagram showing the process and the sequence of robotic motions during an inspection.

The NDE was initiated within the first 2 min of the deposition robot’s retraction to its home position. Figure 8 shows a step-by-step inspection diagram, where at first, the inspection robot’s end-effector approached the wall with a travel speed of 50 mm/s until the position 5 mm above the predicted as-built surface of WAAM was reached.

The second stage in the diagram shows a contact establishment with the WAAM specimen. This was accomplished by an automatic trigger that recognized the robot’s position (5 mm above the expected surface), which was followed by a change of robot speed to an inspection speed (in this work = 2 mm/s) and initiation of FT sensor-driven motion. A command to maintain a constant force of 130 N was sent to the inspection robot from LabVIEW via RSI; thus, the *Z*-axis position correction was no longer managed by a LabVIEW motion framework, but the kinematics corrections were calculated and applied by the KRC. The force applied to the component was set to a maximum force given by the FT sensor operational limit. 

During the descending of the inspection robot on the surface of WAAM, the LabVIEW program was set to wait for 2 s, before sending coordinates of the next position. This “wait” command enabled the inspection robot to position itself on the surface with the required set stable force and without further freedoms in the X-Y plane that could result in inconsistent contact with a specimen.

Stage 3 of the inspection was initiated by sending coordinates of the next target position (in this scenario = the end of an inspection, +300 mm in the *X*-axis direction) and enabling encoded FMC data acquisition. The FMC data were acquired while the inspection robot traveled along the path with a steady force by correcting its *Z*-axis position to maintain a given force value with the experimental wall. 

Once the end of the path was reached, the termination of the inspection was triggered by the change of the inspection robot’s *Z*-axis targeted position. This was given as the *Z*-axis target position offset larger than 5 mm above the predicted WAAM surface. The trigger was used to disable the sensor-driven motion and the ultrasound data acquisition. The process was concluded by retracting the inspection robot to its home position according to the path planning. 

The inspection volume from the experimental wall was set to 300 mm, therefore the time elapsed to inspect the component equaled 150 s with an additional approximate 60 s that included the approach to the specimen and the robot retraction back to a home position. It is worth mentioning, that the entire inspection took significantly less time than the period set for a dwelling (9 min), which complemented the objectives required for the in-process NDE of WAAM in this scenario. The total number of positions encoded FMC frames acquired was 200, giving a sample density of 1.5 per mm (sampling frequency = 0.75 Hz).

### 4.3. Ultrasound Data Post-Processing: TFM Imaging and C-Scan

After the completion of the in-process NDE, the ultrasound data were processed using a SAFT-TFM algorithm described in [22]. The TFM frames (B-Scan) were computed for a 25 mm × 19 mm region at 6 pixels/mm resolution, which is compatible with the 2 dB Amplitude Fidelity criterion of ASME V [36]. This window represented an internal volume of the desired component between the baseplate and a region 2 mm beneath the surface or just above the interface of layers 5 and 6, where potential defects would be expected. Moreover, this work was focused on the detection of tungsten tubes, therefore there was no interest in detecting and analyzing possible generated true defects from the WAAM process, since the calibration for these defects has not yet been developed.

To achieve a full C-scan, the computation was initiated by the ultrasound surface reconstruction using a SAFT surface imaging and surface finding algorithm. Afterward, the curves representing the WAAM surface contours were augmented into the 3-layer adaptive TFM algorithm to produce the TFM frames before their normalization. Normalizing all the frames used to construct the C-scan aided to visualize the entire image on the same dB scale. Using the raw unnormalized frames, the C-scan was formed by populating a new 2-dimensional array’s columns with maximum detected amplitudes from all TFM frame’s columns from n number (*n* = 200) of TFM frames. 

The size of the C-scan presented in this paper was set to 150 × 200 pixels (Number of pixels in the horizontal axis of the TFM frame × the number of frames). The resulting C-scan image was normalized and plotted on a dB scale from the peak amplitude to an averaged noise level (0 to −12 dB), giving the best visual contrast between a signal from tungsten tubes and interference from the base noise levels. 

## 5. In-Process Inspection Results and Discussion 

**Figure 9:**
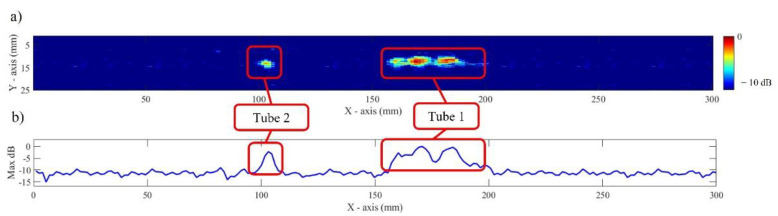
Results showing: (**a**) C-scan obtained from computed TFM frames and (**b**) extracted maximum amplitude along *X*-axis.

In this section, the results of an in-process NDE are presented and discussed. The outcome of the in-process inspection is depicted in Figure 9a, where the signal from Tube 1 and Tube 2, with an internal diameter of 1.0 mm was successfully detected. At a first glance, stronger signal levels are observed from a longitudinally placed 30 mm long Tube 1. Noteworthy that a matching signal extension of approximately 30 mm along the inspection travel direction is also well noticeable. Tube 2, embedded in the traverse direction, shows visually weaker signal strength where the energy from the tube is represented by a concentrated signal in the centre of the corresponding frames approximately 100 mm from the inspection start point. 

Following a visual analysis of the results, a maximum amplitude along an *X*-axis was presented in Figure 9b. Based on this plot, a Signal-to-Noise ratio (SNR) of up to 12 dB was achieved from the scanning of Tube 1 while an SNR of 10 dB was seen for Tube 2. Considering the dry-coupling condition, these SNR values were found sufficiently high for the indications to stand out from the background noise and be readily detected by the operator.

Further analysis shows signal strength variations from Tube 1 signal along the scan path where an SNR drop of only 4 dB was observed. This local signal strength loss can be associated with the possible changes to the contact quality between the rubber tyre and the non-flat wave-like surface profile of WAAM. This means the signal strength propagating into the specimen was fluctuating with the varying profile of WAAM. Further losses of SNR, especially for Tube 2, could be associated with a lack of compensation for the thermal gradient that affects an ultrasound wave velocity during propagation, as also pointed out in [12,37]. This means the image signal amplitude is negatively affected due to the loss of focusing precision during TFM image forming. 

## 6. Conclusions and Future Work

In this paper, a design and demonstration of a novel multi-robot cell for WAAM and ultrasound in-process NDE was presented. The architecture, based on two robotic industrial manipulators featuring a deployed plasma arc WAAM process and high-temperature PAUT roller-probe was introduced along with a software control package, merging manufacture and NDE into a single continuous process. 

The in-process NDE capability was demonstrated on a dry-coupled ultrasound in-process inspection of the Ti-6Al-4V WAAM wall with embedded tungsten tube reflectors, with an internal diameter of 1.0 mm. Using the FMC data acquisition, a C-scan image of the experimental wall was computed by deploying a SAFT-TFM package. The results of the in-process inspection showed successfully detected embedded tubes, with distinguishable SNR of up to 12 dB. 

Therefore, this work demonstrated the ability to detect defects just after the point of generation, which can pave the way for possible in-process repair processes to be deployed in the future. It can also be concluded that the presented research enables the further amplification of WAAM benefits by the deployment of flexible and automated NDE. 

The future development is aimed at improving the speed of image forming, by the employment of graphics processing units. Moreover, the performance evaluation is targeted at transduction and automated deployment in various scenarios such as (1) inspection of geometrically complex WAAM components, (2) an investigation of probe deployment while the torch is active elsewhere and (3) at varying robotic NDE speeds. For the in-process defect detection and characterization area, the key aims are based on the development of thermal gradient compensation capabilities, which can further enhance defect detection and accuracy of the inspection approach. 

Lastly, the research aims to develop a defect calibration procedure for various materials and a wide range of natural defects to enable automated defect detection and characterization that can further enhance the automation of the WAAM. 

## Figures and Tables

**Table 1:** Deposition Parameters.

Deposition Parameters	
Current	150 Amps
Wire-feed speed	2.5 m/min
Robot Velocity	0.005 m/s

**Table 2:** Tungsten Tube parameters.

Tungsten Tube	
Tube length	30 mm
Internal diameter	1 mm
Outer diameter	3 mm

**Table 3:** PAUT parameters.

Array Parameters	Value
Element Count	64
Element Pitch	0.5 mm
Element Elevation	10 mm (unfocused)
Element Spacing	0.1 mm
Centre Frequency	5 MHz

## Data Availability

The data can be shared upon reasonable request.
